# Multidimensional landscape of non‐alcoholic fatty liver disease‐related disease spectrum uncovered by big omics data: Profiling evidence and new perspectives

**DOI:** 10.1002/SMMD.20220029

**Published:** 2023-04-17

**Authors:** Zhengyi Zhu, Yuyan Chen, Xueqian Qin, Shujun Liu, Jinglin Wang, Haozhen Ren

**Affiliations:** ^1^ Department of Hepatobiliary Surgery Affiliated Drum Tower Hospital Medical School Nanjing University Nanjing China

**Keywords:** HCC, NAFLD, NASH, omics data, sequencing

## Abstract

Characterized by hepatic lipid accumulation, non‐alcoholic fatty liver disease (NAFLD) is a multifactorial metabolic disorder that could promote the progression of non‐alcoholic steatohepatitis (NASH), cirrhosis, and hepatocellular carcinoma (HCC). Benefiting from recent advances in omics technologies, such as high‐throughput sequencing, voluminous profiling data in HCC‐integrated molecular science into clinical medicine helped clinicians with rational guidance for treatments. In this review, we conclude the majority of publicly available omics data on the NAFLD‐related disease spectrum and bring up new insights to inspire next‐generation therapeutics against this increasingly prevalent disease spectrum in the post‐genomic era.

## Graphical abstract

Development of omics technologies, such as RNA‐seq, metabonomics, lipidomics, ST RNA‐seq, ATAC‐seq, microbiome‐seq, SC RNA‐seq and others, promotes voluminous profiling data in NASH‐related disease into clinical medicine.

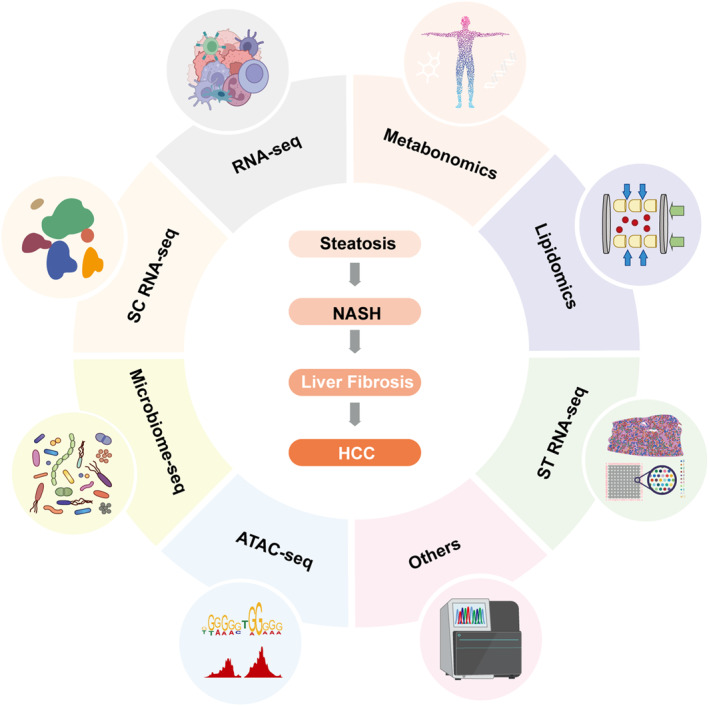

1

**Key points**

- We review the research progress in the field of the non‐alcoholic fatty liver disease (NAFLD) disease spectrum.
- We focus on the results of high‐throughput sequencing techniques from NAFLD to hepatocellular carcinoma (HCC).
- We discuss the clinical application prospect and future direction of big data generated by multi‐omics technology.

## 2 INTRODUCTION

**FIGURE 1:**
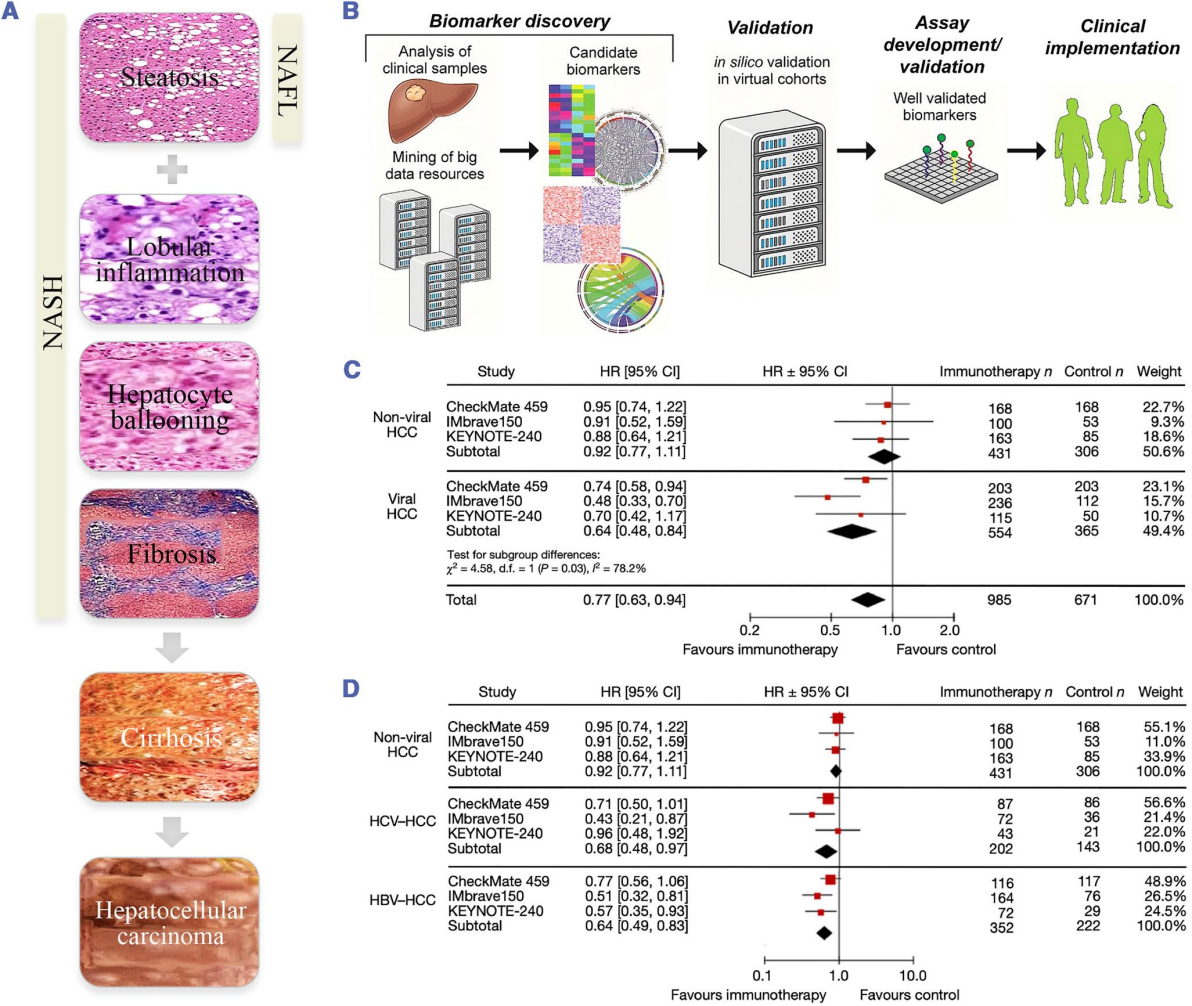
Progression of the NAFLD disease spectrum. (A) The progressive stages of NAFLD. Reproduced with permission.^3^ Copyright 2017, Taylor & Francis. (B) Recent advances in omics technologies transmit voluminous profiling data in HCC‐integrated molecular science that has been applied to clinical medicine. Reproduced with permission.^4^ Copyright 2017, Elsevier Inc. (C and D) A meta‐analysis of 1656 patients was performed to compare immunotherapy responses between nonviral (NASH and alcohol intake) and viral (HBV and HCV) groups. Reproduced under terms of the CC‐BY license.^5^ Copyright 2021, The Authors, published by Springer Nature. HBV, hepatitis B virus; HCC, hepatocellular carcinoma; HCV, hepatitis C virus; NAFLD, non‐alcoholic fatty liver disease; NASH, non‐alcoholic steatohepatitis.

Representing the hepatic manifestation of obesity and metabolic syndrome, non‐alcoholic fatty liver disease (NAFLD) influences over 25% of the world's population, of whom up to one quarter may have non‐alcoholic steatohepatitis (NASH).^1^ Defined as the pathological accumulation of triglycerides in hepatocytes due to etiologies other than excessive alcohol consumption, the NAFLD disease spectrum ranges from simple hepatic steatosis to NASH and liver cirrhosis with the potential to develop into hepatocellular carcinoma (HCC) in a minor proportion of cases^2^, ^3^ (Figure 1A). Given the increased global morbidity of obesity and Type 2 diabetes, hepatic steatosis with inflammation and injury by definition called NASH^6^ is recognized as an increasingly important underlying etiology of HCC.^7^ Estes et al. predicted that NASH‐associated mortality will increase more than double by 2030 due to the aging and increasing world population.^8^ Recently, a meta‐analysis of multiple phase III trials comprising 1656 patients with unresectable HCC demonstrated that anti‐PD1 or anti‐PD‐L1 treatments were less effective in NASH‐driven HCC compared with viral HCC^5^ (Figure 1C,D). However, owing to insufficient data to draw reliable conclusions, international consensus guidelines do not consider etiology for the clinical management of HCC patients nowadays. This highlights the importance of elucidating the intra‐tumor heterogeneity and underlying mechanisms in NAFLD‐HCC progression to provide greater clinical benefits.

Benefiting from recent advances in omics technologies, such as high‐throughput sequencing (HTS), voluminous profiling data in HCC‐integrated molecular science into clinical medicine helped clinicians with rational guidance for treatments^4^ (Figure 1B). Produced by detection methods, including genomics, transcriptomics, proteomics, metabolomics, microbiomics, etc., big data could provide a global perspective complementing molecular mechanisms and detect imperceptible high‐level information patterns. Multi‐domain systematically collated public omics data repositories, such as the International Cancer Genome Consortium and The Cancer Genome Atlas, have fueled the developments of cancer research,^9^ and have spawned a large range of analytic pipelines to assess data. Despite various publicly accessible resources suitable for HCC research, the surfeit of datasets associated with NAFLD, NASH, and NAFLD‐related fibrosis or HCC remained dispersed and non‐curated on the basis of a tremendous volume of sequencing data.

Recent years have witnessed great enthusiasm for big data‐driven methodologies, which have considerably enriched our understanding of the molecular and genetic basis of the NAFLD‐related disease spectrum. In this review, we give a summary of recent technological advances in HTS‐based biological insights, focusing on the contribution of big omics data in exploring the patterns of disease progression from NAFLD to HCC. Moreover, the potential for translating the wealth of genetic data into the clinical implementation of new therapeutic designs and diagnostic biomarkers will be recapitulated. In summary, we will conclude the majority of publicly available omics data on the NAFLD‐related disease spectrum and bring up new insights to inspire next‐generation therapeutics against this increasingly prevalent disease spectrum in the post‐genomic era.

## 3 LARGE‐SCALE BULK SEQUENCING DATA UNRAVELING MOLECULAR MECHANISMS

As a robust and mainstay technique, transcriptomics of bulk tissue samples could provide a broad and deep understanding of the complexity of the specific disease and unravel the molecular profiles correlated with the disease progression. Accompanied by advances in emerging data analysis techniques, bulk RNA‐sequencing (RNA‐seq) of patients with the NAFLD disease spectrum largely improves the translatability of candidate therapeutic targets and may afford important clinical and translational implications for clinicians.

**FIGURE 2:**
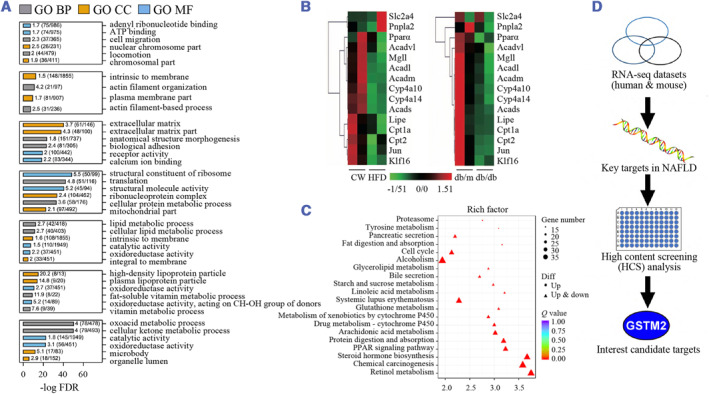
Large‐scale bulk sequencing data unraveling the molecular mechanisms. (A) The Gene Ontology (GO) analysis of the rat CDAA model. Reproduced under terms of the CC‐BY license.^13^ Copyright 2020, The Authors, published by Springer Nature. (B) Heatmap showing the expression of hepatic KLF16 and other genes regulating lipid metabolism. Reproduced with permission.^14^ Copyright 2021, The Authors, published by BMJ Publishing Group Ltd. & British Society of Gastroenterology. (C) Heatmap showing the increased arachidonic acid metabolism. Reproduced with permission.^12^ Copyright 2021, Elsevier B.V. (D) Schematic diagram of the screening strategy of potential targets in NAFLD by the analysis of RNA‐seq data. Reproduced with permission.^15^ Copyright 2022, The Authors, published by Elsevier B.V. CDAA, choline‐deficient, L‐amino acid‐defined diet; NAFLD, non‐alcoholic fatty liver disease.

Because of the high incidence of Type 2 diabetes and severe overweight, it is essential to uncover NAFLD's specific pathogenesis and mechanism. By performing RNA‐seq on liver samples from overweighed individuals, Gerhard et al. observed that pathways associated with cytokine–cytokine receptor interaction, PI3K‐AKT signaling pathway, focal adhesion, and extracellular matrix‐receptor interaction are enriched in specimens with interlobular inflammation and advanced fibrosis of NAFLD patients compared with normal tissues.^10^ Ghallab et al. analyzed the sequence related to translation events in NAFLD progression via RNA‐seq to characterize the time‐dependent alternations of the transcriptomics landscape and calculated the degree to which transcriptomic profiles of mice resemble human NAFLD.^11^ With the large proportion of perturbations in the NAFLD liver compared to the healthy control, transcriptome analysis showed that the majority of dysregulated protein‐coding genes were linked with glycolipid metabolism^12^ (Figure 2C). The expression profiles and features of circular RNA (circRNA) in NAFLD mice were analyzed by Yuan, and a total of 93 dysregulated circRNAs were identified.^16^ These in‐depth molecular characterizations of hepatic steatosis depicted the map of disease initiation and revealed specific molecular signatures that may lead to the progression of NAFLD.

In the design of a few studies regarding NASH, RNA‐seq was used to screen the core candidate genes like Krüppel‐like factor 16 (KLF16)^14^ (Figure 2B) and glutathione S‐transferase mu 2 (*Gstm2*)^15^ (Figure 2D). RNA‐seq analysis performed on NASH mice demonstrated that increased arachidonic acid metabolism enhances ferroptosis in NASH, substantiated by the accumulation of lipid reactive oxygen species (ROS), morphological change of mitochondria, and the increased cell death.^17^ A panoramic analysis of sequencing data on NASH provided insights into the roles of proinflammatory cytokines and chemokines, inflammasome pathways, trained immunity enzymes, and lipid peroxidation in the development of NASH.^18^ Comparable results are observed in the rat choline‐deficient, L‐amino acid‐defined diet (CDAA) model^13^ (Figure 2A). By conducting RNA‐seq analysis on 60 adult liver samples with different degrees of NASH, Atanasovska et al. found a series of long non‐coding RNAs (lncRNAs) related to NASH phenotypes, especially inflammation, and identified a novel intergenic lncRNA‐designated lncTNF, which regulates the NF‐κB signaling pathway.^19^

Meanwhile, Pantano et al. performed total RNA‐seq on NAFLD patients throughout the spectrum of the fourth stage of fibrosis, namely cirrhosis, and demonstrated highly relevant genes, including COL1A2, EFEMP2, FBLN5, and THBS2.^20^ In addition, pro‐apoptotic pathways, bipotent hepatocyte markers, and cholangiocyte precursors were found to increase in the fibrosis stage.^20^ The transcriptomic analysis revealed that a specific gene expression signature, including *Slc41a*, *Fabp5*, *Igdcc4*, and *Mthfd1l*, was identified in NASH‐HCC, along with significantly enriched pathways related to macrophage infiltration, angiogenesis, and stemness features.^21^ These studies provided a major increment in information regarding the molecular mechanisms underlying the NAFLD disease spectrum, and mutual verification between the source data may elicit unprecedented and compelling insights into the landscape of this spectrum.

## 4 SINGLE‐CELL TRANSCRIPTOMIC SIGNATURES OF THE NAFLD DISEASE SPECTRUM

**FIGURE 3:**
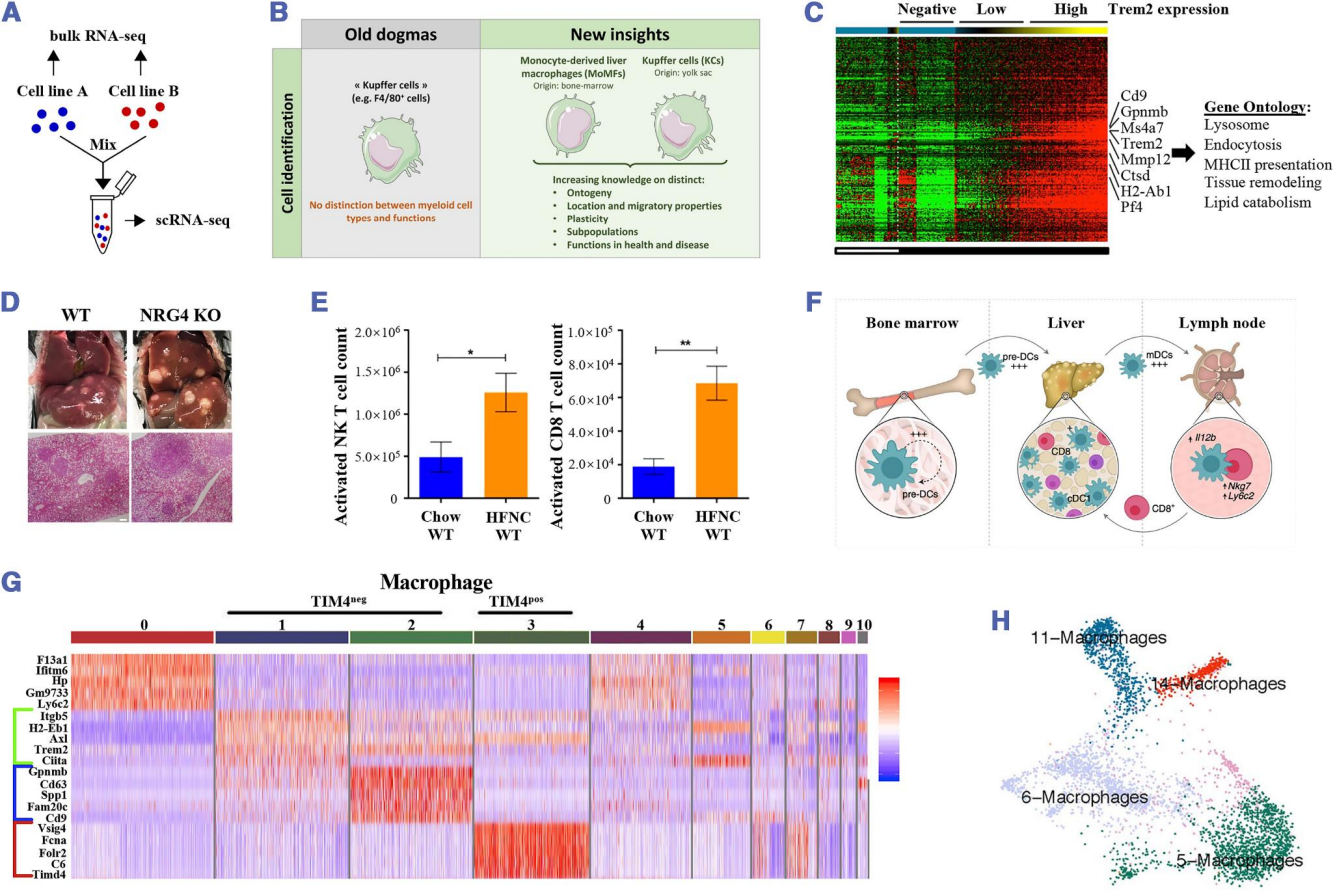
Single‐cell transcriptomic signatures of the NAFLD disease spectrum. (A) Generation of a scRNA‐seq dataset with a known cellular composition. Reproduced under terms of the CC‐BY license.^22^ Copyright 2019, The Authors, published by Springer Nature. (B) Old dogmas versus new insights on liver macrophages. Reproduced with permission.^23^ Copyright 2019, The Authors, published by Wiley Periodicals, Inc. (C) Heatmap representation of the macrophage gene expression according to the Trem2 expression. Reproduced with permission.^24^ Copyright 2019, Elsevier. (D) The exhaustion of cytotoxic CD8+ T cells, linking NASH to the occurrence of hepatic carcinoma. Reproduced with permission.^25^ Copyright 2022, Elsevier Inc. (E) The increased number of infiltrating NKT and CD8+ T cells in the livers of mice with NASH. Reproduced with permission.^26^ Copyright 2017, The Authors, published by Wiley Periodicals, Inc. (F) Model of the systemic cDC activity in NASH. Reproduced with permission.^27^ Copyright 2021, The Authors, published by Springer Nature. (G) Macrophage heterogeneity during NASH was defined. Reproduced with permission.^28^ Copyright 2021, The Authors, published by Elsevier Inc. (H) UMAP projection of the four macrophage clusters. Reproduced under terms of the CC‐BY license.^29^ Copyright 2022, The Authors, published by Springer Nature. NAFLD, non‐alcoholic fatty liver disease; NASH, non‐alcoholic steatohepatitis.

Distinct from conventional bulk RNA sequencing, single‐cell RNA sequencing (scRNA‐seq) is a potent deep molecular profiling technique that enables genome‐wide transcriptomes with single‐cell granularity^22^ (Figure 3A). Here, we will highlight the latest findings related to cellular communication circuits in the NAFLD/NASH spectrum and discuss the concrete roles of different cell subsets participating in the progression of NAFLD or NAFLD‐derived tumors.

Liver‐residential Kupffer cells (KCs) are the most abundant macrophage population in the human body and function as critical immunologic sentinels in the liver. Recently, unbiased large‐scale techniques, like scRNA‐seq, have revealed the complexity of macrophage polarization that is beyond the recognition of pro‐inflammatory (M1) and anti‐inflammatory (M2) phenotypes^23^ (Figure 3B). For the characterization of cell‐type‐specific transcriptional features in NASH, Fred et al. conducted the scRNA‐seq analysis in 19,627 cells derived from liver specimens of 10 patients and demonstrated the differences in abnormal activation of macrophage populations in NASH^29^ (Figure 3H). Applying high‐dimensional approaches of scRNA‐seq and single‐cell proteomics, Blériot et al. identified two distinct subsets of embryonic KCs based on CD206 and ESAM expressions, termed KC1 (CD206^lo^ESAM‐) and KC2 (CD206^hi^ESAM+).^30^ Evidence showed that the KC2 population was increased in steatosis and could induce oxidative stress via CD36.^30^ In addition, Xiong et al. isolated non‐parenchymal cells from NASH mouse livers and uncovered the NASH‐associated macrophages (NAMs) marked by *Trem2*, which are associated with the severity of NASH^24^ (Figure 3C). Meanwhile, single‐cell data from Daemen's study pointed out that these NAMs, namely *Trem2* ^pos^ KCs expressing *Gpmnb* and *Cd9*, are in fact monocyte‐derived macrophages (MdMs)^28^ (Figure 3G). By employing scRNA‐seq to identify the heterogeneity of myeloid cells in NASH livers, Krenkel et al. observed a unique inflammatory phenotype characterized by decreased calprotectin (S100A8/A9) in macrophages and dendritic cells.^31^ Furthermore, Zhang et al. found that tumor‐associated macrophage (TAM)‐like macrophages are induced in NASH livers, accompanied by the exhaustion of cytotoxic CD8+ T cells, linking NASH to the occurrence of hepatic carcinoma, as revealed by single‐cell transcriptomic studies^25^ (Figure 3D).

During NASH, single‐cell profiling also suggested that an abundance of hepatic conventional dendritic cells (cDCs), especially type 1 cDCs expressing chemokine X‐C receptor 1 (XCR1), shows a remarkable increase in the liver and is associated with NASH worsening^27^ (Figure 3F). Previous studies showed that patients with NASH have striking hepatic infiltrations of NKT and CD8+ T‐cells^26^ (Figure 3E). Further, by using scRNA‐seq, Dudek et al. detected the hepatic accumulation of a conserved and expanded CD8+ T cell population marked by CXCR6, PD‐1, and granzyme B, which triggered the auto‐aggression of killing hepatocytes via Fas‐FasL interactions.^32^ Specific subclusters of activated hepatic stellate cells (HSCs) featured by the expression of ACTA2 and RBP1 were identified to contribute to the fibrosis of NASH through collagen deposition.^29^ Single‐cell secretome gene analysis identified HSCs to secrete stellakines associated with chronic liver injury.^24^ Similarly, Terkelsen et al. revealed critical HSC genes that act as constitutive markers of advanced fibrosis in NASH patients at a single‐cell resolution via transcription dynamics of hepatic sinusoid‐associated cells.^33^

Combined with modern single‐cell analysis techniques, these studies provided key evidence for us to gain a deep understanding of the cellular and molecular mechanisms of the NAFLD disease spectrum and could be repeatedly used for further analyses to determine novel potential therapeutic targets for the treatment.

## 5 GUT MICROBIOTA DYSBIOSIS REVEALED BY THE FECAL MICROBIOME

Nowadays, high‐throughput technologies represented by the 16S ribosomal RNA (rRNA) gene sequencing approach are widely used to profile intestinal bacterial communities, while multiple studies have determined that abnormalities of the gut‐liver axis homeostasis could lead to the development of the NAFLD disease spectrum.^34^ Here, we provided an overview of the insights that have been gained into the microbiome signatures of NAFLD and NAFLD‐derived HCC and focused on deciphering the underlying metabolic disorders in the disease course.

**FIGURE 4:**
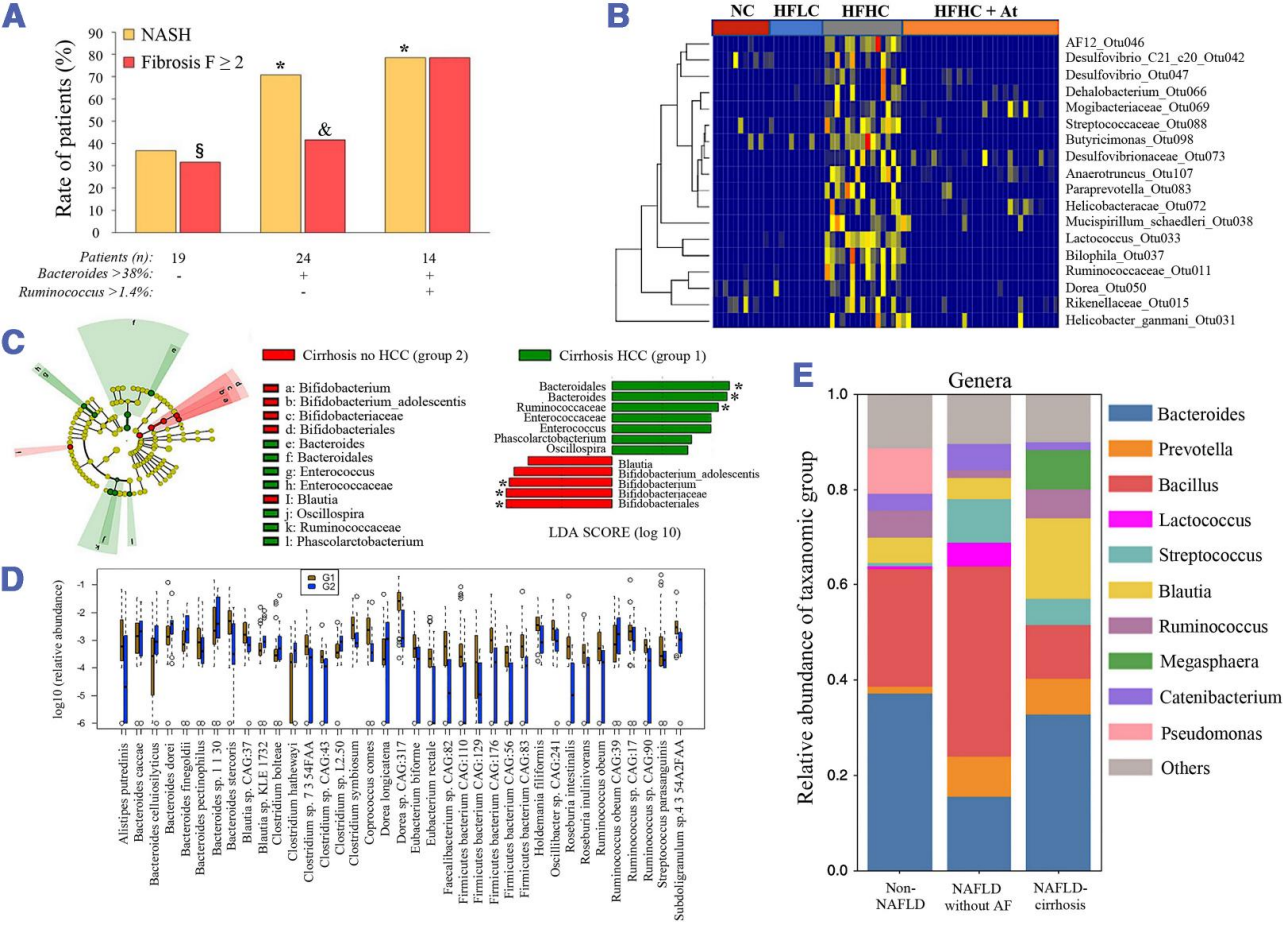
Gut microbiota dysbiosis revealed by the fecal microbiome. (A) Rate of patients with NASH or fibrosis F2 according to *Bacteroides* and *Ruminoccocus* abundance (*P: vs. first subgroup; §P: vs. second subgroup; & P: vs. third subgroup). Reproduced with permission.^39^ Copyright 2016, John Wiley and Sons. (B) Heatmap plot of the bacteria in stool of mice fed with NC, HFLC, and HFHC with or without atorvastatin treatment. Reproduced with permission.^40^ Copyright 2021, The Authors, published by BMJ Publishing Group Ltd. (C) Linear discriminant effect size (LEfSe) analysis between cirrhotic patients with HCC. Reproduced with permission.^42^ Copyright 2019, John Wiley and Sons. (D) Relative abundances of species among the different liver fibrosis groups. Reproduced with permission.^37^, ^38^ Copyright 2017, Elsevier. Copyright 2019, Elsevier. (E) The gut microbiome composition of non‐NAFLD controls, NAFLD without AF, and NALFD‐cirrhosis probands. Reproduced under terms of the CC‐BY license.^41^ Copyright 2019, The Authors, published by Springer Nature. NAFLD, non‐alcoholic fatty liver disease; NASH, non‐alcoholic steatohepatitis.

Increasing evidence suggested that compared to healthy individuals, more phylum *Bacteroidetes* and fewer *Firmicutes* were observed in the fecal microbiota of NAFLD patients, while Gram‐negative bacteria were prevalent in NAFLD as well.^35^ Notably, Raman et al.'s research pointed out that several members of phylum *Firmicutes*, including *Dorea*, *Robinsoniella*, and *Roseburia*, are overrepresented in NAFLD, while one special member of *Firmicutes* termed *Oscillibacter* is underrepresented in the fecal microbiome of NAFLD patients.^36^ By using whole‐genome shotgun sequencing, Loomba et al. determined that the increased Gram‐negative *Proteobacteria* (including *E. coli*) phylum and the decreased Gram‐positive *Firmicutes* phylum were detected along with disease progression from mild or moderate NAFLD to advanced NASH fibrosis^37^, ^38^ (Figure 4D). In addition, *Bacteroides* abundance was identified to be independently correlated with the severity of NASH lesions, while the abundance of *Ruminococcus* showed a significant correlation with NASH‐driven fibrosis^39^ (Figure 4A). By 16S rRNA gene amplicon analysis, Caussy et al. revealed that the family Enterobacteriaceae and the genera *Streptococcus* and *Gallibacterium* were more abundant in NAFLD‐related cirrhosis and confirmed that more Gram‐negative microbes would appear in the advanced fibrosis stages of the NAFLD spectrum^41^ (Figure 4E). By 16S rRNA sequencing, Zhang et al. determined that the gut microbiota compositions, including *Mucispirillum*, *Desulfovibrio*, *Anaerotruncus*, and *Desulfovibrionaceae*, increased sequentially along stages from NAFLD to HCC, while the proportions of *Bifidobacterium* and *Bacteroides* exhibited significant decreases in the NAFLD‐HCC formation^40^ (Figure 4B). Liquid chromatography‐mass spectrometry (LC‐MS) metabolomic analysis showed an increase in the taurocholic acid (TCA) concentration in serum, while serum 3‐indole propionic acid (IPA) was found to be depleted in NAFLD‐derived HCC.^40^ Ponziana et al. also explored the features of the gut microbiota in NAFLD‐related HCC and identified that *Bacteroides*, *Oscillospira*, and *Enterococcus* were enriched in adults with HCC when compared to patients with liver cirrhosis^42^ (Figure 4C).

Although previous studies have provided an abundance of evidence regarding the gut microbiome as a causal factor of the NAFLD spectrum, it is worth noting that endogenous or exogenous factors could dynamically influence the gut bacterial communities,^43^ and interactions between the gut microbiome and NAFLD‐related pathophysiological processes still await further investigations.

## 6 METABOLOMICS OR LIPIDOMICS ANALYSES DEPICTING METABOLIC TRAITS

Metabolomics refers to the comprehensive profiling concerned with small‐molecule metabolites, such as amino acids, fatty acids, and carbohydrates, while lipidomics is defined as a portion of metabolomics mainly regarding cellular lipids. Given that the hallmark of the NAFLD disease spectrum is the intracellular accumulation of lipids, especially triglycerides, investigations on the characterization of metabolic traits play pivotal roles in revealing the mechanisms underlying pathogenesis.

**FIGURE 5:**
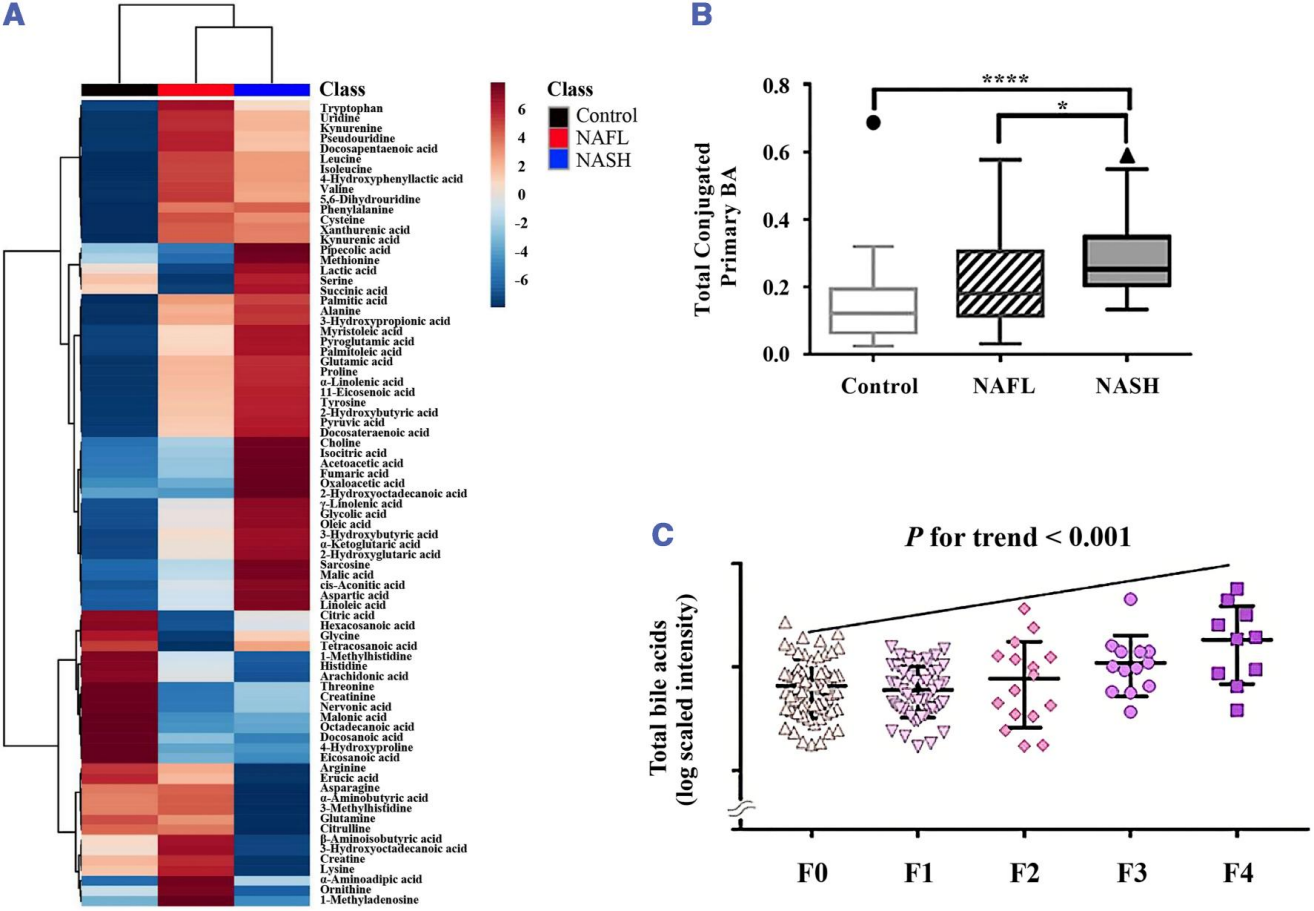
Metabolomics or lipidomics analyses depicting metabolic traits. (A) Hierarchical clustering heatmaps showed the normalized plasma metabolite levels of NAFL, NASH patients, and the control group. Reproduced under terms of the CC‐BY license.^44^ Copyright 2022, The Authors, published by MDPI. (B) Total conjugated primary bile acids in control, NAFL, and NASH groups. Reproduced with permission.^45^ Copyright 2018, John Wiley and Sons. (C) Scatter dot plot of log‐scaled intensity of total bile acids in different liver fibrosis stages. Reproduced with permission.^46^ Copyright 2019, John Wiley and Sons.

Utilizing the gas chromatography (GC)‐MS/MS and LC‐MS/MS techniques, metabolomics analysis was performed on plasma samples of NAFLD patients and the control group, and a total of 79 metabolites were detected in Ji et al.'s study^44^ (Figure 5A). Amino acids (AAs; including alanine, valine, glutamic acid, tyrosine, and α‐aminoadipic acid), organic acids (OAs; including 2‐hydroxybutyric acid, 3‐hydroxy propionic acid, and α‐ketoglutaric acid), fatty acids (FAs; including myristoleic acid, palmitoleic acid, α‐linolenic acid, and docosapentaenoic acid), and kynurenic acid were remarkably increased in NAFLD patients^44^ (Figure 5A). By performing the quantitative lipidomic analysis on liver biopsies with NAFLD or NASH, Chiappini et al. determined that the lipid characteristics of NASH are associated with aberrant regulation of the fatty acid synthesis pathway.^47^ Specifically, the concentrations of saturated fatty acids (SFAs), including palmitate acid and stearate acid as well as free cholesterol, sphingolipids, glycerophospholipids, and eicosanoids were increased during the progression of NASH.^48^

Bile acid toxicity has been unraveled to participate in the pathogenesis of NAFLD. By measuring bile acids in the plasma of patients, Puri et al. found an increase in total primary bile acids and a decrease in secondary bile acids in NASH^45^ (Figure 5B). In addition, Caussy et al. reported that elevated primary conjugated bile acid and decreased unconjugated bile acid and unconjugated cholyl and chenodeoxycholyl conjugates were observed along the progression of liver fibrosis stages in NASH patients^46^ (Figure 5C). Despite great progress in the field of metabolic rearrangement in the NAFLD disease spectrum, targeted therapies are still scarce and require further studies to implement these findings into clinical care.

## 7 MULTIFACETED PORTRAITS GENERATED BY OTHER OMICS TECHNIQUES

Besides the above‐mentioned omics technologies, the continued development and utilization of high‐throughput technologies, such as epigenomics or proteomics, have also contributed greatly to improving our understanding of the complex mechanisms underlying the NAFLD disease spectrum and ultimately identifying effective treatments for this prevalent disease.

**FIGURE 6:**
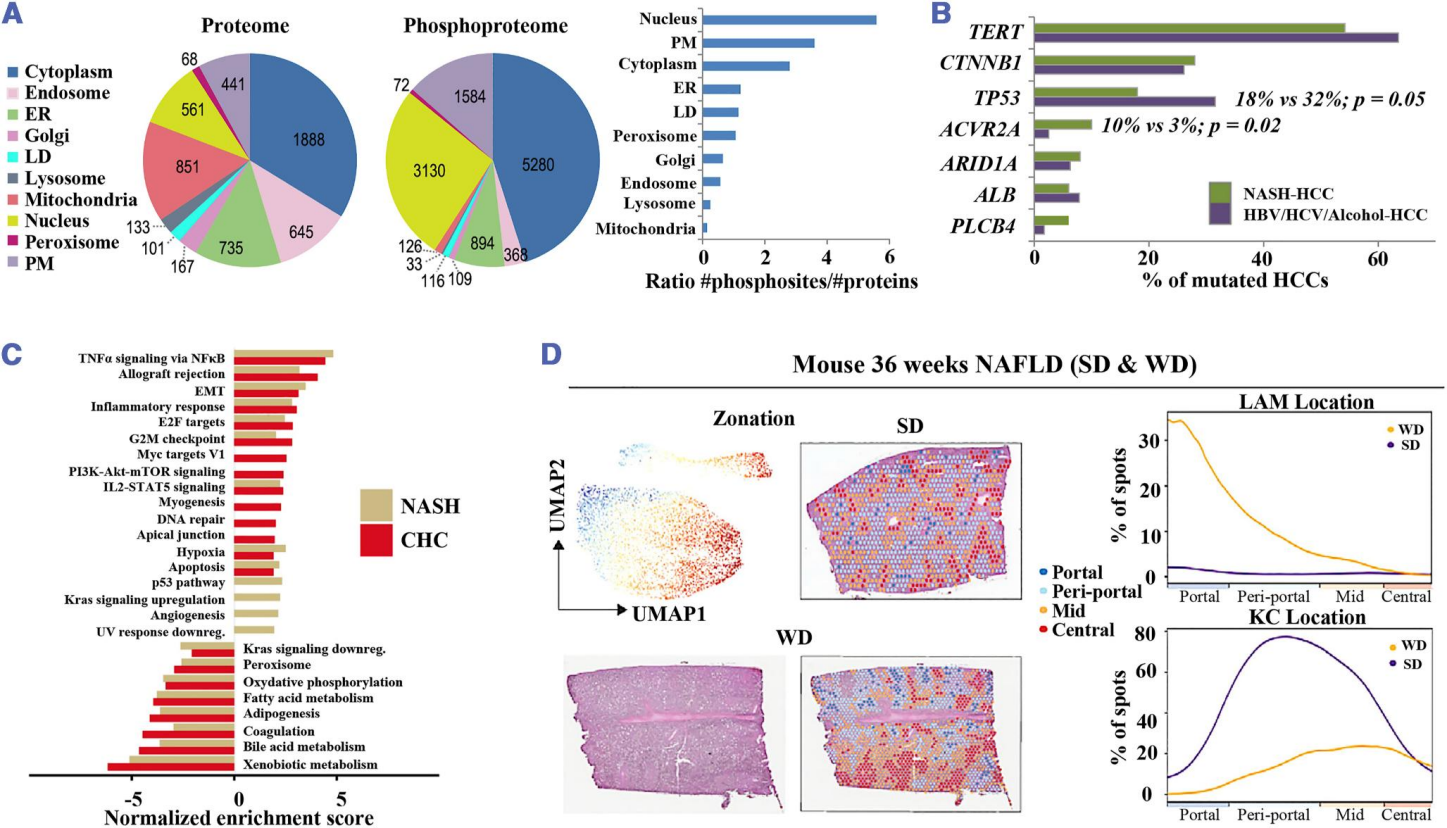
Multifaceted portraits generated by other omics techniques. (A) Characterization of subcellular organelle proteome and organelle‐specific phosphorylation in mouse liver. Reproduced with permission.^50^ Copyright 2018, Elsevier Inc. (B) Bar plot of mutation frequencies in NASH‐derived HCC compared to HCC samples from other etiologies. Reproduced with permission.^51^ Copyright 2021, Elsevier. (C) Hallmark pathways significantly enriched for H3K27ac modifications in NASH and CHC compared with control patient samples. Reproduced with permission.^52^ Copyright 2021, The Authors, published by BMJ Publishing Group Ltd. (D) Zonation pattern and H&E staining (left) and LAM and KC location (right). Reproduced under terms of the CC‐BY license.^53^ Copyright 2022, The Authors, published by Elsevier Inc. HCC, hepatocellular carcinoma; NAFLD, non‐alcoholic fatty liver disease; NASH, non‐alcoholic steatohepatitis.

Proteomic techniques have emerged as potent tools for biomarker discovery, enabling the identification of protein fingerprints in blood or tissues that hold potential as disease markers.^49^ Organellar proteomics and phospho‐proteomics showed the levels and cellular distributions of 6000 liver proteins and 16,000 phosphopeptides in the progression of NASH in Krahmer's study^50^ (Figure 6A). Yuan et al. identified 132 upregulated proteins and 84 downregulated proteins in NAFLD compared to patients with metabolic healthy obesity.^54^ Proteomic analyses showed that NASH leads to high plasma levels of the hepatokine TSK.^55^ By analyzing 48 patient samples, Niu et al. used plasma proteome profiling technology and identified elevated PIGR in both NAFLD and cirrhosis and a correlation of DPP4, ANPEP, TGFBI, PIGR, and APOE with the diseases.^56^ The SOMAscan proteomics technology was employed to measure 1305 blood proteins in a study group of 113 individuals, revealing that 97 proteins with varying functions showed disparities in expressions between patients with advanced and early NAFLD fibrosis.^57^ Sveinbjornsson's study used proteomic data and uncovered 18 sequence variants associated with fatty liver and 4 with cirrhosis, 16 putative causal genes implicated in lipid metabolism, and multiple plasma proteins involved in NAFLD disease pathogenesis.^58^ These findings have opened new avenues for the development of diagnostic and therapeutic approaches for NAFLD and related liver diseases. Continued research in this area is critical for the advancement of the field and the translation of proteomic discoveries into clinical applications.

Epigenetic modifications have emerged as a critical area of investigation in understanding the pathogenesis of the NAFLD disease spectrum as they have been shown to play a significant role in regulating gene expression.^59^ Utilizing the chromatin immunoprecipitation followed by sequencing (ChIP‐seq) profile based on ChIP mentation, Jühling et al. detected the H3K27ac epigenetic markers on active promoters and enhancers in NASH patients and found that genes correlated with xenobiotic, bile acid, and fatty acid metabolism, including coagulation, adipogenesis, and oxidative phosphorylation, showed markedly a reduced level of H3K27ac, while genes regarding TNF‐α signaling via NF‐κB, epithelial‐to‐mesenchymal transition (EMT), and inflammatory response demonstrated increased H3K27ac levels^52^ (Figure 6C). Luo et al. found that m6A methylation modifications tend to be positively correlated with NAFLD by using methylated RNA immunoprecipitation sequencing (MeRIP‐seq).^60^ Meanwhile, it is worth noting that partial sequencing datasets on Epigenetic modifications of miRNAs, lncRNAs, and circRNAs can often be found in the literature on RNA sequencing.

Previous studies have identified various genetic factors that contribute to the development and progression of the NAFLD disease spectrum. Anstee's study compared the genetic profiles of NAFLD cases and controls by Genome‐Wide Association Study (GWAS) and identified that PNPLA3, TM6SF2, HSD17B13, and PYGO1 are risk factors for the full histological spectrum of NAFLD.^61^ To identify genetic variants associated with NAFLD, Ghodsian et al. performed a genome‐wide meta‐analysis and identified five potential susceptibility sites (GCKR, TR1B1, MAU2/TM6SF2, APOE, and PNPLA3) and the potential effects of LPL expression and FTO genotypes on NAFLD.^62^ Whole exome sequencing (WES) identified that TERT, CTNNB1, TP53, and ACVR2A are mutated frequently in NASH‐derived HCC compared to HCC samples of other etiologies^51^ (Figure 6B). Similarly, by conducting WES in 425,671 participants from the UK Biobank, Jamialahmadi et al. identified two novel genetic variants in GPAM and APOE that are strongly correlated with steatosis and liver damage.^63^ These findings demonstrate the potential of genetic profiling to improve our understanding of this disease spectrum and may lead to the identification of new therapeutic targets.

Spatial proteogenomics is a cutting‐edge technology that allows for the simultaneous visualization of gene expression patterns and histological features in intact tissue sections, providing insights into the spatial organization of cell types and their interactions within complex biological systems.^53^ By spatial proteogenomics atlas, Guilliams et al. determined that lipid‐associated macrophages (LAMs) are recruited to the steatotic regions preferentially in the livers of patients with NAFLD and uncovered the restricted space and conservative ligand‐receptor pairs between KCs^53^ (Figure 6D). However, research in this field remained scarce and required further investment from the scientific community.

Currently, remarkable breakthroughs in omics techniques and data analysis have provided intensive insights into our understanding of the progression of the NAFLD disease spectrum and facilitated the identification of potential therapeutic targets.

## 8 CONCLUSIONS

By comprehensively and systematically studying the molecular pathology of the NAFLD disease spectrum, the rapidly evolving high‐throughput omics techniques have produced a comprehensive snapshot of patients and greatly facilitated clinical research advances. By understanding current perspectives on transcriptomics, proteomics, epigenomics, microbiome, metabolomics, and other aspects, we summarize the specific contributions of these technologies to understanding the mechanisms of the NAFLD disease spectrum (Table 1), which may provide support for future diagnostic and therapeutic research.

**TABLE 1:** Molecular mechanisms underlying the NAFLD disease spectrum using omics analyses.

Analysis	Literature	Stage	Specimens	Molecular mechanisms^a^
RNA‐seq	Ghallab et al.,^11^ 2021	NAFLD	Human, mouse	Time‐dependent alternations of the transcriptomics landscape during NAFLD progression
RNA‐seq	Xu et al.,^12^ 2021	NAFLD	Mouse	Dysregulated glycolipid metabolism
RNA‐seq	Yuan et al.,^16^ 2020	NAFLD	Mouse	93 dysregulated circRNAs
RNA‐seq	Sun et al.,^14^ 2021	NASH	Human, mouse	Screening of core candidate gene KLF16
RNA‐seq	Lan et al.,^15^ 2022	NASH	Human	Screening of core candidate gene Gstm2
RNA‐seq	Li et al.,^17^ 2020	NASH	Mouse	Increased arachidonic acid metabolism enhances ferroptosis in NASH
RNA‐seq	Drummer et al.,^18^ 2021	NASH	Human, mouse	Proinflammatory cytokines and chemokines, inflammasome pathways, trained immunity enzymes, and lipid peroxidation
RNA‐seq	Atanasovska et al.,^19^ 2021	NASH	Human	Identification of lncRNAs related to NASH phenotypes
RNA‐seq	Gerhard et al.,^10^ 2018	NASH, fibrosis	Human	Cytokine‐cytokine receptor interaction, PI3K‐AKT signaling pathway, focal adhesion, and extracellular matrix‐receptor interaction
RNA‐seq	Pantano et al.,^20^ 2021	Fibrosis	Human	COL1A2, EFEMP2, FBLN5, and THBS2; pro‐apoptotic pathways, bipotent hepatocyte markers, and cholangiocyte precursors
RNA‐seq	Simoni‐Nieves et al.,^21^ 2021	NASH‐HCC	Mouse	*Slc41a*, *Fabp5*, *Igdcc4*, and *Mthfd1l*; pathways related to macrophage infiltration, angiogenesis, and stemness features
scRNA‐seq, single‐cell proteomics	Blériot et al.,^30^ 2021	NAFLD	Mouse	Distinct subsets of embryonic KCs (KC1 and KC2) based on CD206 and ESAM expressions
scRNA‐seq	Xiong et al.,^24^ 2019	NASH	Mouse	Identification of NAMs marked by Trem2
scRNA‐seq	Daemen et al.,^28^ 2021	NASH	Mouse	NAMs (Trem2^pos^ KCs expressing *Gpmnb* and *Cd9*) are MdMs
scRNA‐seq	Krenkel et al.,^31^ 2020	NASH	Mouse	Unique inflammatory phenotype characterized by decreased calprotectin in macrophages and dendritic cells
scRNA‐seq	Deczkowska et al.,^27^ 2021	NASH	Mouse	An increased abundance of hepatic cDCs (especially type 1 cDCs expressing XCR1) is associated with NASH worsening
scRNA‐seq	Dudek et al.,^32^ 2021	NASH	Mouse	CD8+ T cell population marked by CXCR6, PD‐1, and granzyme B
Single‐cell secretome	Xiong et al.,^24^ 2019	NASH	Mouse	HSCs secrete stellakines associated with liver injury
scRNA‐seq	Fred et al.,^29^ 2022	NASH, fibrosis	Human	Abnormal activation of macrophage populations; subclusters of activated HSCs featured by ACTA2 and RBP1 expressions
scRNA‐seq	Terkelsen et al.,^33^ 2020	NASH, fibrosis	Mouse	Critical HSC genes that act as constitutive markers of advanced fibrosis in NASH patients
scRNA‐seq	Zhang et al.,^25^ 2022	NASH, NASH‐HCC	Mouse	Induction of TAM‐like macrophages in NASH livers is linked to the occurrence of HCC
16S rRNA‐seq	Wang et al.,^35^ 2016	NAFLD	Human	Increased *Bacteroidetes*, decreased *Firmicutes*, and prevalence of Gram‐negative bacteria
16S rRNA‐seq	Raman et al.,^36^ 2013	NAFLD	Human	Overrepresented *Dorea*, *Robinsoniella*, and *Roseburia*; underrepresented *Oscillibacter*
Whole‐genome shotgun sequencing	Loomba et al.,^37^, ^38^ 2017	NASH, fibrosis	Human	Increased *Proteobacteria*, decreased *Firmicutes* with disease progression
16S rRNA‐seq	Boursier et al.,^39^ 2016	NASH, fibrosis	Human	*Bacteroides*: Correlated with the severity of NASH; *Ruminococcus*: Correlated with NASH‐driven fibrosis
16S rRNA‐seq	Caussy et al.,^41^ 2019	Fibrosis	Human	The family Enterobacteriaceae, the genera *Streptococcus* and *Gallibacterium*, and Gram‐negative microbes
16S rRNA‐seq	Zhang et al.,^40^ 2021	NAFLD‐HCC	Human, mouse	Increased *Mucispirillum*, *Desulfovibrio*, *Anaerotruncus*, and Desulfovibrionaceae; decreased *Bifidobacterium* and *Bacteroides*
16S rRNA‐seq	Ponziana et al.,^42^ 2019	NAFLD‐HCC	Human	Enriched *Bacteroides*, *Oscillospira*, and *Enterococcus*
GC‐MS/MS, LC‐MS/MS	Ji et al.,^44^ 2022	NAFLD	Human	79 metabolites
Lipidomics	Chiappini et al.,^47^ 2017	NAFLD, NASH	Human	Aberrant regulation of the fatty acid synthesis pathway
Lipidomics	Musso et al.,^48^ 2018	NASH	Human	SFAs: Palmitate acid, stearate acid; free cholesterol, sphingolipids, glycerophospholipids, and eicosanoids
LC/MS	Puri et al.,^45^ 2018	NASH	Human	Increased total primary bile acids; decreased secondary bile acids
LC/MS	Caussy et al.,^46^ 2019	NASH, fibrosis	Human	Elevated primary conjugated bile acid; decreased unconjugated bile acid, unconjugated cholyl, and chenodeoxycholyl conjugates
Proteomics	Yuan et al.,^54^ 2020	NAFLD	Human	132 upregulated proteins and 84 downregulated proteins
Proteomics	Sveinbjornsson et al.,^58^ 2022	NAFLD	Human	22 sequence variants, 16 putative causal genes, and multiple plasma proteins
MeRIP‐seq	Luo et al.,^60^ 2021	NAFLD	Mouse	m6A methylation modifications
GWAS	Anstee et al.,^61^ 2020	NAFLD	Human	PNPLA3, TM6SF2, HSD17B13, and PYGO1
Genome‐wide meta‐analysis	Ghodsian et al.,^62^ 2021	NAFLD	Human	Five potential susceptibility sites: GCKR, TR1B1, MAU2/TM6SF2, APOE, and PNPLA3
WES	Jamialahmadi et al.,^63^ 2021	NAFLD	Human	Two novel genetic variants in GPAM and APOE
Spatial proteo‐genomics	Guilliams et al.,^53^ 2022	NAFLD	Human, mouse	LAMs recruited to steatotic regions; restricted space and conservative ligand‐receptor pairs between KCs
Proteomics, phospho‐proteomics	Krahmer et al.,^50^ 2018	NASH	Mouse	Levels and cellular distributions of 6000 liver proteins and 16,000 phosphopeptides
Proteomics	Xiong et al.,^55^ 2019	NASH	Mouse	High plasma levels of the hepatokine TSK
ChIP‐seq	Jühling et al.,^52^ 2021	NASH	Human	Reduced H3K27ac: Xenobiotic, bile acid, and fatty acid metabolism‐related genes; increased H3K27ac: TNF‐α signaling, EMT, and inflammatory response‐related genes
Proteomics	Niu et al.,^56^ 2019	NAFLD‐fibrosis	Human	DPP4, ANPEP, TGFBI, PIGR, and APOE
Proteomics	Luo et al.,^57^ 2021	NAFLD‐fibrosis	Human	Identification of 97 proteins with varying functions
WES	Pinyol et al.,^51^ 2021	NASH‐HCC	Human	Mutated TERT, CTNNB1, TP53, and ACVR2A

Abbreviations: HCC, hepatocellular carcinoma; NAFLD, non‐alcoholic fatty liver disease; NASH, non‐alcoholic steatohepatitis.

^a^ Versus corresponding control group.

RNA‐seq technology is a powerful sequencing‐based technology that enables researchers to study gene expression with high resolution and reproducibility, detect novel transcripts and alternative splicing events, and identify splice isoforms, sequence variation, and fusion transcript genome wide.^64^ Nowadays, RNA‐seq analysis of liver samples from overweight individuals has revealed gene expression signatures associated with inflammation, fibrosis, apoptotic pathways, and stemness features in NAFLD patients. However, RNA sequencing technology suffers specific potential problems, such as potential biases, limitations in sequencing depth, and the inability to detect RNA modifications and spatial information.^65^ By offering a static snapshot of gene expression, scRNA‐seq provides a means to appreciate cellular heterogeneity within a population.^66^ Recent studies using scRNA‐seq have shed light on cellular communication circuits in the NAFLD disease spectrum and the roles of different cell subsets in the progression of the disease. For example, liver‐resident KCs have been shown to have complex macrophage polarization, and scRNA‐seq analysis has demonstrated differences in macrophage activation in NASH.^29^ Non‐parenchymal cells from NASH mouse livers have been isolated and marked by Trem2, and single‐cell data showed that these cells are monocyte‐derived macrophages.^24^, ^28^ Other studies have shown that tumor‐associated macrophage‐like cells are induced in NASH livers and are linked to hepatic carcinoma.^25^ Single‐cell profiling has also revealed the increased abundance of hepatic conventional dendritic cells in NASH and the accumulation of a specific population of CD8+ T cells in the liver.^26^ Additionally, activated hepatic stellate cells have been identified as contributing to fibrosis in NASH through collagen deposition, and the single‐cell secretome gene analysis has shown that they secrete stellakines associated with chronic liver injury.^24^, ^29^ These studies provide a deeper understanding of NAFLD's cellular and molecular mechanisms and can be used to determine potential therapeutic targets.

Notably, MS‐based proteomics enables in‐depth qualitative and quantitative characterization of the proteome of any organism, which is essential for understanding the underlying cell biology, physiology, and biochemistry.^67^ For instance, the proteomic analysis showed that NASH leads to high levels of TSK,^55^ and disparities in expression were revealed between patients with advanced and early NAFLD fibrosis using the SOMAscan proteomics technology.^57^

In addition, we present a comprehensive review of the present knowledge of the gut microbiome and its correlation with the development and progression of the disease spectrum that arises from NAFLD. High‐throughput 16S rRNA gene amplicon sequencing has emerged as a robust method for investigating microbial communities.^68^ Previous literature suggested that NAFLD patients have an increased abundance of *Bacteroidetes* and Gram‐negative bacteria^35^ and decreased abundance of Gram‐positive *Firmicutes* ^37^, ^38^ with some studies pointing to the overrepresentation of specific members of the *Firmicutes* phylum and depletion of *Oscillibacter*.^36^ Notably, researchers often complement 16S rRNA gene sequencing with other technologies or alternative methods, such as metagenomics, to overcome its limitations and enhance the accuracy and comprehensiveness of the results. For example, there have been studies linking gut microbiota to changes in serum metabolites, such as increased taurocholic acid and decreased 3‐indole propionic acid.^40^ In NAFLD patients, GC‐MS/MS and LC‐MS/MS techniques revealed increased amino acids, organic acids, and fatty acids,^44^ and lipidomic analysis identified aberrant regulation of the fatty acid synthesis pathway.^47^ Bile acid toxicity also contributes to NAFLD pathogenesis.^45^, ^46^ However, targeted therapies are still limited and further studies are necessary for clinical implementation.

In addition to the aforementioned, recent studies have applied various other high‐throughput omics technologies to explore the molecular basis of the NAFLD disease spectrum. ChIP‐seq detected changes in H3K27ac epigenetic markers in NASH patients,^52^ and MeRIP‐seq showed a positive correlation between m6A methylation modifications and NAFLD.^60^ Genetic risk factors and mutations associated with NAFLD were also identified using GWAS^61^ and WES.^51^ The spatial proteogenomics atlas showed that lipid‐associated macrophages are recruited to steatotic regions in the livers of NAFLD patients.^53^ Overall, these omics techniques and data analysis have provided valuable insights into NAFLD progression and potential therapeutic targets.

With the development of sequencing technologies, applying multi‐omics techniques to the same biological system facilitates understanding the information flow of underlying diseases and interpreting the data in a holistic manner in the context of biological networks and molecular interactions.^69^ The method involves integrating data from multiple high‐throughput omics technologies, including genomics, transcriptomics, proteomics, epigenomics, and metabolomics, to reveal a more complete and accurate picture of the underlying molecular mechanisms of the disease.^70^ For instance, Atabaki‐Pasdar et al. used multi‐omics and clinical data from the IMI DIRECT cohort to develop models predicting the liver fat content in European‐ancestry adults with or at risk of type 2 diabetes.^71^ Wood et al. developed a multi‐component classifier for NAFLD in adults with extreme obesity, using proteomic, genomic, and phenotypic variables, and the final classifier achieved an AUC of 0.935, indicating better predictive power than a single‐domain analysis and suggesting the potential for improved biomarkers and diagnostic tests for hepatic steatosis in obese individuals.^72^ Involving RNA‐Seq and proteomics analysis of liver samples, as well as proteomics analysis of plasma, Veyel's study used a multi‐omics approach to identify plasma biomarkers for NASH.^13^ Similarly, Ægidius et al. used a multi‐omics approach to characterize tissue biopsies from NASH mice, revealing distinct phenotypes, correspondences between mRNA and protein levels, and key cell types involved in NASH pathogenesis.^73^ In summary, in addition to improving predictive power, multi‐omics composite biomarker groups may also capture more biological complexity of disease pathogenesis and progression than traditional markers that typically focus on a single aspect of the NAFLD‐related disease spectrum.

In all, various high‐throughput sequencing technologies have the potential to aid in the development of predictive models, improved biomarkers, and diagnostic tests for the NAFLD‐related disease spectrum and may ultimately inform more effective clinical treatment strategies.

## AUTHOR CONTRIBUTIONS

Haozhen Ren and Jinglin Wang conceived the conceptualization and designed the paper. Zhengyi Zhu and Yuyan Chen wrote the paper. Zhengyi Zhu, Yuyan Chen, Xueqian Qin, and Shujun Liu contributed to the revision of the paper.

## CONFLICT OF INTEREST STATEMENT

The authors declare no conflict of interest.
